# Screening for autism spectrum disorders: state of the art in Europe

**DOI:** 10.1007/s00787-014-0555-6

**Published:** 2014-06-10

**Authors:** Patricia García-Primo, Annika Hellendoorn, Tony Charman, Herbert Roeyers, Mieke Dereu, Bernadette Roge, Sophie Baduel, Filippo Muratori, Antonio Narzisi, Emma Van Daalen, Irma Moilanen, Manuel Posada de la Paz, Ricardo Canal-Bedia

**Affiliations:** 1 Institute of Rare Diseases Research (Instituto de Investigación de Enfermedades Raras, IIER), Carlos III Institute of Health, Consortium for Biomedical Research in Rare Diseases (Centro de Investigación Biomédica en Red de Enfermedades Raras-CIBERER), Madrid, Spain; 2 Department of Educational Sciences, Utrecht University, Utrecht, The Netherlands; 3 Department of Psychology, King’s College London’s Institute of Psychiatry, London, England; 4 Department of Experimental Clinical and Health Psychology, Ghent University, Ghent, Belgium; 5 Laboratoire Octogone, EA 4156, Université de Toulouse le Mirail, Toulouse, France; 6 Department of Child Neurology and Psychiatry, IRCCS Stella Maris Foundation, Pisa, Italy; 7 Department of Child Psychiatry, University and University Hospital of Oulu, Oulu, Finland; 8 Department of Child and Adolescent Psychiatry, University Medical Centre, Utrecht, The Netherlands; 9 Faculty of Education, University Institute of Community Integration (Instituto Universitario de Integración en la Comunidad-INICO), University of Salamanca, Salamanca, Spain; 10 Institute of Rare Diseases Research (Instituto de Investigación de Enfermedades Raras, IIER), Carlos III Institute of Health, Madrid, Spain; 11 Instituto de Salud Carlos III, IIER-Pab 11, Monforte de Lemos, 5, 28029 Madrid, Spain

**Keywords:** Autism, Screening, Methods, Early, Detection, Review, Europe

## Abstract

A large number of studies have reported on the validity of autism spectrum disorder (ASD) screening procedures. An overall understanding of these studies’ findings cannot be based solely on the level of internal validity of each, since screening instruments might perform differently according to certain factors in different settings. Europe has led the field with the development of the first screening tool and first prospective screening study of autism. This paper seeks to provide an overview of ASD screening studies and ongoing programmes across Europe, and identify variables that have influenced the outcomes of such studies. Results show that, to date, over 70,000 children have been screened in Europe using 18 different screening procedures. Differences among findings across studies have enabled us to identify ten factors that may influence screening results. Although it is impossible to draw firm conclusions as to which screening procedure is most effective, this analysis might facilitate the choice of a screening method that best fits a specific scenario, and this, in turn, may eventually improve early ASD detection procedures.

## Introduction

In the past few decades, many studies have documented behavioural manifestations of ASD during the first 2 years of life [1–5]. Nevertheless, there is still a large delay between the first parental concerns, the first consultation, and the age at which the diagnosis is made [6–8]. Early identification and subsequent intervention lead to a better prognosis for the child. Intervention may prevent secondary developmental disturbances [9–11] and reduce family stress [6, 12] and societal costs [13–15]. Thus, there is a need to develop methods and instruments for early identification of ASD.

The first attempt to develop a prospective screening instrument for ASD was made in Europe by Baron-Cohen and his colleagues in the UK with the Checklist for Autism in Toddlers (CHAT; [16]). In over 20 years that have elapsed since the CHAT was presented, much progress has been made, with more than 20 ASD screening instruments currently available at international level (Table 1). It remains to be seen, however, whether current screening instruments fulfil the criteria for large-scale implementation [41]. Although a number of studies have shown that early ASD screening is feasible, there are still several issues to be addressed. Experts have noted that few screening instruments are well-evaluated and that it is important for both clinical and research purposes to collect more structured, in-depth information on existing screening procedures [42].

**Table 1:** ASD screening tools

Screening tool (long name)	Short name	Admin. time (min)	Admin. age (months)	Admin. method^b^	Items	Sensitivity	Specificity
Level 1^a^							
Checklist for Autism in Toddlers [16, 17]	CHAT	5–10	18	Parent + clinician rated	9 + 5	0.18–0.38	0.98–1.0
Social Communication Questionnaire [18]	SCQ	15–20	36–82	Parent rated	40	0.74	0.54
Modified-Checklist for Autism in Toddlers [19]	M-CHAT	5–10	18–30	Parent rated	23	0.87	0.99
Quantitative Checklist for Autism in Toddlers [20]	Q-CHAT	5	16–30	Parent rated	25	–	–
Communication and Social Behaviour Scale-Infant and Toddlers Checklist [21]	CSBS-DP	5–10	16–30	Parent rated	24	–	–
Early Screening Autistic Traits Questionnaire [22]	ESAT	10	14–15	Parent + child care worker	14	–	–
First Year Inventory [23]	FYI	10	12	Parent rated	59	–	–
Checklist for Early Signs of Developmental Disorders [24]	CESDD			Child care worker rated	12		
Autism Observation Scale for Infants [1]	AOSI	10	6–1	Clinician rated	18	0.84	0.98
Young autism and other developmental disorders checkup tool [25]	YACHT-18	10	18	Clinician rated	18	0.82	0.86
The Social Attention and Communication Study [26]	SACS	5	8, 12, 18, 24	Clinician rated	15	0.83	0.99
Joint attention-observation schedule [27]	JA-OBS	5–10	20–48	Child Nurse Rated	5	0.86	–
Level 2^a^							
Developmental Behaviour Checklist-primary care version [28]	DBC-ES	5–10	18–48	Parent rated	96	0.83	0.48
Screening tool for autism in 2 years old [29]	STAT	20	24–35	Child care worker rated	12	0.83	0.86
Screening for infants with developmental deficits and/or autism [30]	SEEK	30–40	8	Parent + clinician rated	9 + 28	–	–
Pervasive Developmental Disorders Rating Scale [31]	PDDRS	60	>12	Parent rated	51	–	–
Autistic behavioural indicators instrument [32]	ABII	30	24–72	Clinician rated	18	–	–
Autism Behaviour Checklist [33]	ABC	15	>36	Parent rated	57	0.58	0.76
Childhood Rating Scale [34]	CARS	15–20	>24	Clinician rated	15	0.92–0.98	0.85
Autism detection in early childhood [35]	ADEC	12	12	Parent or nurse rated	16	0.79–0.94^a^	0.88–1.00^a^
Baby and Infant Screen for Children with Autism Traits [36–39]	BISCUIT	15	17–37	Parent rated	42	0.84	0.86
Three-item direct observation screen test [40]	TIDOS	5	18–60	Clinician rated	3	0.95	0.85

^a^Level 1 = population-based screening; level 2 = ASD specific screening tool after developmental delay risk confirmation at a routine developmental surveillance

^b^Clinician = usually paediatrician or primary care physician

Novel screening instruments have been developed in Europe over the past decade, including the Early Screening of Autistic Traits in The Netherlands (ESAT; [22, 43]) and the Checklist for Early Signs of Developmental Disorders in Belgium (CESDD; [24]). Screening instruments have also been translated, culturally adapted and tested in countries other than those where they were originally developed, e.g. the Modified-Checklist for Autism in Toddlers (M-CHAT; [19]) in Spain [44] and in Sweden [27]. Other European countries, such as France, Italy and Finland, are currently engaged in evaluating other screening procedures for which results are still to be published.

To date there has been little exchange of information among researchers across Europe regarding the details of the screening procedures used and the difficulties encountered during screening. There are very few studies that report on rigorous direct comparisons of different screening procedures in similar circumstances [45, 46]. Rather than developing new screening instruments, a careful look at previous and ongoing ASD screening programmes in Europe might instead provide key insight for improving current and future screening procedures. Examination of the same screening procedures in different samples and contexts may be a good way of identifying strengths and weaknesses. In addition, evaluating the effectiveness of different adaptations of existing screening procedures may contribute to identifying the factors that influence screening outcomes.

The COST Action ‘Enhancing the Scientific Study of Early Autism’ (ESSEA) has brought together a group of European researchers who use screening instruments to identify ASD prospectively at an early age [47]. One of the aims of this collaboration is to identify which screening instruments perform best in a given context. Current health care, social and educational systems across Europe vary greatly in terms of expertise and capacity to identify children with ASD at a young age, often leading to marginalisation and disparities between social classes on the mean age of diagnosis [48, 49]. The positive effects of early screening to reduce racial/ethnic and socio-economic status inequalities in age of first diagnosis are promising [50] although these effects have to be further explored [51]. Indeed, there are no European ASD screening guidelines. Even within individual countries, societal, demographic and service factors might affect how screening works, and yet these factors do not tend to be well described in studies. The purpose of this paper is thus to describe the procedures used in ASD screening studies conducted across Europe, and to summarise the respective factors and methodological issues which might have influenced the results of the different studies.

## Current situation of ASD screening studies in Europe

**Fig. 1:**
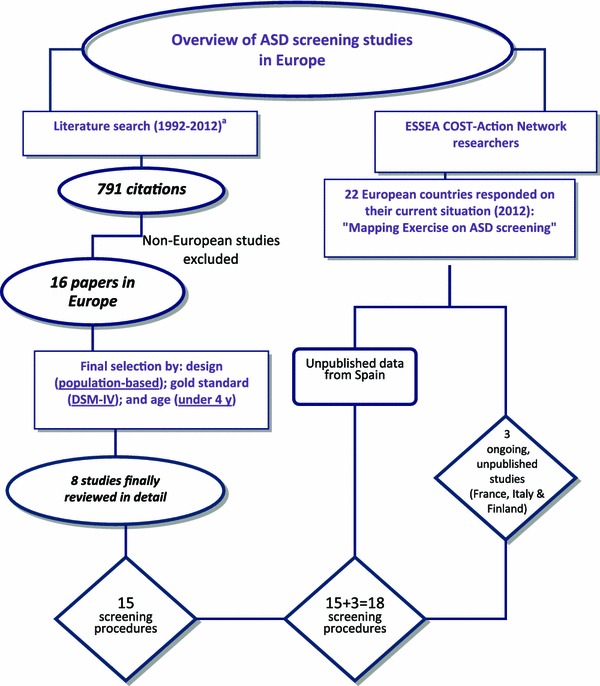
Searching strategy for ASD screening studies in Europe. *Letter a* indicates new literature review and consultation of ESSEA-COST members have been carried out just before March 2014 but none new ASD screening studies in Europe have been published either communicated to main authors apart from the already included

To obtain a complete picture of the status of ASD screening in Europe, we used a two-pronged search process (See Fig. 1). A search of the scientific literature was made covering the PubMED and PsycINFO databases and using the following search terms: ‘autism’ OR ‘autism spectrum disorder’ AND ‘screening’ or ‘identification’ or ‘detection’, with “1992–2012 Pub-date” and “English language” as advanced filters. This search retrieved over 700 citations. Perusal of the titles, authors and abstracts of these citations to discard any study that had been not undertaken in Europe, yielded a net total of 16 papers. When reviewing these papers, the following additional selection criteria were applied for their final inclusion: (a) design: population based; (b) participants: children under the age of 4 years at first screening and with no prior diagnosis of developmental delay (no school-age tool); and, (c) gold-standard diagnostic procedure: DSM-IV-TR criteria for pervasive developmental disorders (PDDs), also known as autism spectrum disorders (ASDs) [52] and the autism diagnostic observation schedule (ADOS; [24]). The reference lists of all relevant studies were checked to identify any additional publications. Using these selection criteria, papers reporting screening at school age, as in Finland [53, 54] and the UK [55–60], were excluded. Similarly excluded were the study conducted in Ireland [61] because it did not use the DSM-IV as standard diagnostic procedure, and the study undertaken by Allison et al. [20] because it was not population based. Eight studies reporting 15 screening procedures for young pre-schoolers with ASD in Europe were retained for review.

Secondly, researchers within the ESSEA COST action network were approached to ascertain whether there might be any other ongoing, as yet unpublished screening programmes. As a result, a further three screening procedures were identified in France, Italy and Finland, and preliminary data were incorporated into this review, leading to a total of 18 different screening procedures. Where published studies failed to provide data on sensitivity, specificity, positive predictive value (PPV) and negative predictive value (NPV), these measures were estimated from the data, if available (to be taken with caution since different protocol adaptations are used). In addition, all main authors were asked to provide clarification regarding the procedures and results of their studies, as well as verification of the information to be included in this paper. An overview can be found in Table 2.

**Table 2:** Overview of European screening studies

Setting and users	Screening procedure	Study sample and results^a^	Comments
United Kingdom—South East Thames region Primary health care practitioner to parents	CHAT (high + medium risk) + CHAT (high + medium risk)	*N* = 16.235, *M*_age_ = 18.7 (1.1) PPV = 0.59; NPV = 1.00; Se. = 0.21; Sp. = 1.00	Extremely low false-positive rate High false-negative rate Specifically, combination of joint attention items + pretend play indicates ASD risk Discriminating protodeclarative acts may be difficult for parents (Baron-Cohen et al. [17], Baird et al. [62])
The Netherlands—Province of Utrecht Well-baby clinics + home Physicians to parents + psychologist to parents	4-item + 14-item ESAT	*N* = 31.724, *M*_age_ = 14.91 (1.37) PPV = 0.25; NPV = *; Se. = *; Sp. = *	High false-positive rate but no TD children At young age, hard to discriminate between ASD and TD/DD At young age, failure to detect higher functioning children/milder ASD variants/children who regress or develop autism later Drop-out because parents not yet willing to cooperate Physicians cautious in referring for ASD Screen-negative cases not followed up (Dietz et al. [43])
The Netherlands—Nijmegen Primary care setting + child psychiatry Primary care worker Primary care worker + parents’ self-administered test Primary care worker + parents’ self-administered test Primary care worker + parents’ self-administered test	Procedure 1: Clinical concern + 14-item ESAT Procedure 2/3: 14-item ESAT + SCQ 11 14-item ESAT + SCQ 15 Procedure 4: 14-item ESAT + CSBS-DP Procedure 5/6: 14-item ESAT + CHAT high risk 14-item ESAT + CHAT high + medium risk	*N* = *, *M*_age_ = PPV = 0.68; NPV = 0.37; Se. = 0.88; Sp. = 0.14 PPV = 0.71; NPV = 0.47; Se. = 0.84; Sp. = 0.28 PPV = 0.79; NPV = 0.48; Se. = 66; Sp. = 0.64 PPV = 0.78; NPV = 0.50; Se. = 0.71, Sp. = 0.59 PPV = 0.97; NPV = 0.37; Se. = 0.18; Sp. = 0.99 PPV = 0.88; NPV = 0.45; Se. = 48; Sp. = 0.87	No screening instrument clearly better than any other in differentiating ASD from non-ASD Trade-off between sensitivity and specificity (F.1) High false-positive rate Explore different cut-offs/item-selection within screening instruments. CHAT not administered in original form, constructed from SCQ and CSBS-DP items Screen-negative cases not followed up: where true sensitivity and specificity could not be calculated, they were calculated with the percentage of children about whom there was already some concern (Oosterling et al. [63])
Belgium—Flanders Child day-care setting + home Child care worker + parents’ self-administered test Child care worker + parents’ self-administered test Child care worker + parents’ self-administered test Child care worker + parents’ self-administered test	Procedure 1: CESDD + 14-item ESAT Procedure 2/3: CESDD + SCQ 11 CESDD + SCQ 15 Procedure 4: CESDD + M-CHAT Procedure 5: CESDD + FYI	*N* = 7.092, *M*_age_ = 16.70 (8.19) PPV = 0.55; NPV = 0.95; Se. = 0.40; Sp. = 0.97 PPV = 0.44; NPV = 0.94; Se. = 0.70; Sp. = 0.84 PPV = 0.83; NPV = 0.91; Se. = 0.43; Sp. = 0.98 PPV = 0.29; NPV = 0.98; Se. = 0.71; Sp. = 0.87 PPV = 1.00; NPV = 0.93; Se. = 0.33; Sp. = 1.00	First screening to include report by child care workers High false-positive rate but many developmental disorders/delays among false positives Low parent compliance rate Adaptation of original screening protocol: no telephone interview included in M-CHAT, ESAT completed by parents alone. (Dereu et al. [24])
Spain—Salamanca and Zamora; Madrid Well-baby clinic + home Parents’ self-administered test + researcher to parents +paediatrician Parents’ self-administered test + paediatrician/nurse to parents through web interface	Procedure 1: M-CHAT + M-CHAT telephone interview(by researchers at Univ. when needed) Procedure 2: M-CHAT + M-CHAT web-based interview	*Salamanca* and *Zamora* *N* = 8,122, *M*_age_ = 20.58 (3.2) PPV = 0.38; NPV = 0.99; Se. = 0.83; Sp. = 0.99 *Madrid* *N* = 2,910, *M*_age_ = 23.14 (4.0) PPV = 0.26; NPV = 0.99; Se. = 0.90; Sp. = 0.99 *N* = 1,402, *M*_age_ = 20.21 (3.0) PPV = 0.50; NPV = 0.99; Se. = 0.67; Sp. = 0.99	Translated and adapted; M-CHAT results similar to original M-CHAT study Explore adaptation with screening instrument, such as web-based interview instead of telephone interview Need for coordination of health services and ASD intervention units in Spain Screen-positive children followed up for 2 years Locating and contacting families for telephone interview proved very time-consuming (García-Primo et al. [64])
Sweden—Gothenburg (Home +) child health centre Nurse Parents’ self-administered test Parents’ self-administered test + nurse	Procedure 1: JA-OBS Procedure 2: M-CHAT (including interview) Procedure 3: M-CHAT (including interview) + JA-OBS	*N* = 3.999, *M*_age_ = 36.00 (no SD reported) PPV = 0.92.5; NPV = .*; Se. = 0.86; Sp. = * PPV = 0.92; NPV = .*; Se. = 0.76; Sp. = * PPV = 0.89.6; NPV = .*; Se. = 0.95.6; Sp. = *	Interview M-CHAT was necessary; many parents had difficulties understanding questions JA-OBS raised nurse awareness about ASD Combining different instruments for professionals and parents is effective. Screen-negative cases not followed up Screening procedure implemented in developmental programme (Nygren et al. [27])
France—Toulouse Well-baby clinic Parents’ self-administered test + professional	M-CHAT + CHAT	*N* = 1,227, *M*_age_ = 24 Preliminary data: TP = 17; TN = 1,192; FN = 1; FP = 17	Difficulty in obtaining participation of professionals Follow-up at 30 and 36 months in order to check the diagnosis status
Italy Paediatrician to parents	M-CHAT + M-CHAT interview by paediatrician directly	*N* = 1,000, *M*_age_ = 24.4 (3.2) Preliminary data: TP = 4; TN = *; FN = *; FP = 8 PPV 0.28	Difficulties in re-screening children with “pass result” in order to find false-negative cases
Finland Nurse + Nurse to parents	Procedure 1(first study attempt): At 18 m.o.:CHAT + ICQ and CBCL +BITSEA	*N* = 200	CBCL (Children’s Behavioural Checklist) No longer ongoing
	Procedure 2(started later): At 12 m.o.: nurse checklist + BITSEA + ICQ + ESAT	*N* = 677	Small sample, no cases with ASD yet Planning modifications in short future

*PPV* positive predictive value,* NPV* negative predictive value,* Se.* sensitivity,* Sp.* specificity,* ASD* autism spectrum disorder,* DD* developmental disorder/delay,* TD* typical development. *M*age in months

^a^Note that the results presented here need to be taken with caution since some of the tools have been used in unusual or adapted conditions and for that reason cannot be considered as the unique psychometric properties of the too

* Number is unknown and could neither be extracted from the literature nor calculated from the data

**Fig. 2:**
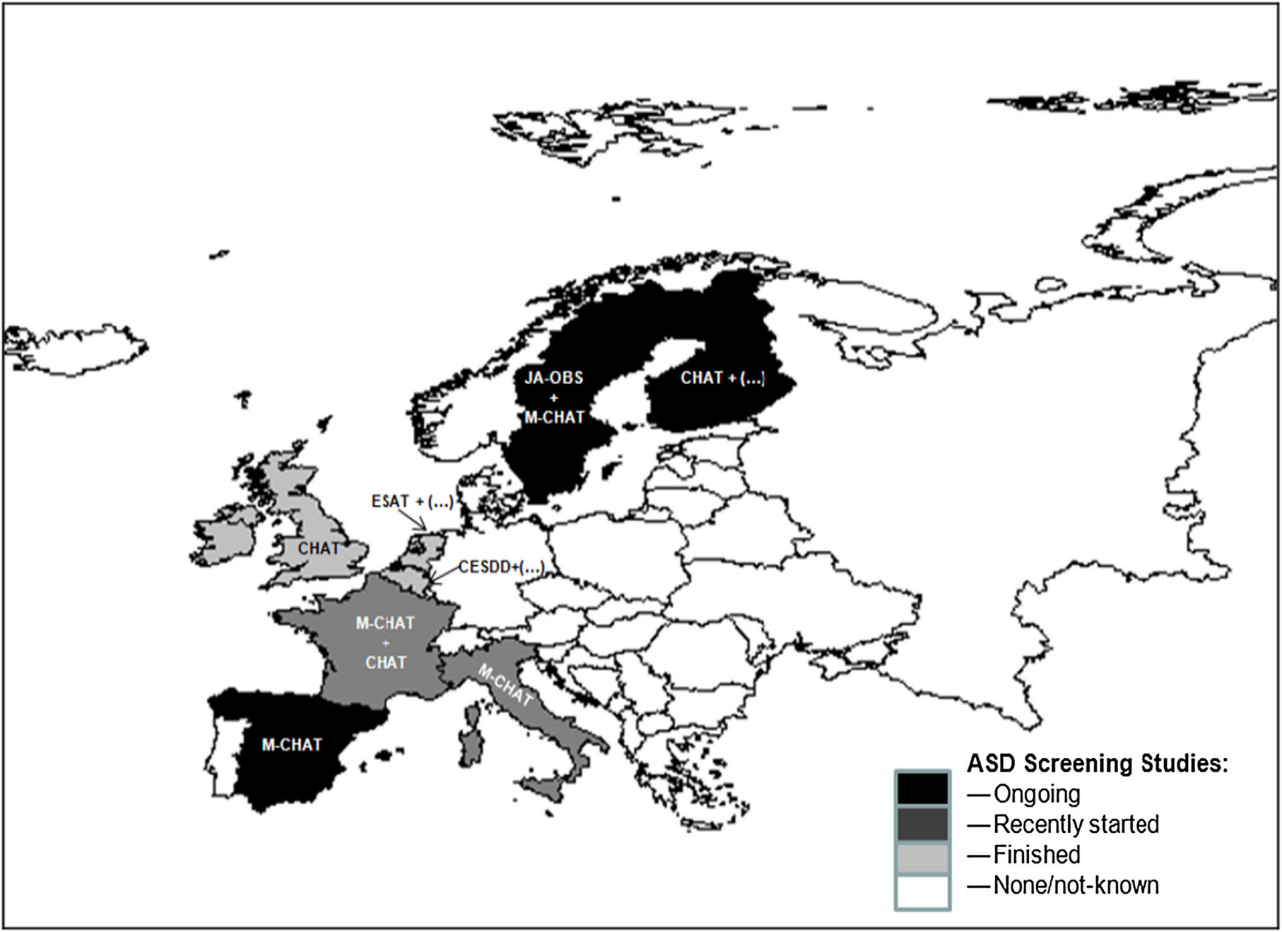
Map of the situation of ASD European screening studies in 2012–2013

This table shows information on the number of completed and ongoing ASD screening studies across Europe. Over 70,000 children have been screened in Europe to date. Nine of the 28 European Union Member States (32 %) have conducted or are conducting ongoing ASD screening studies (although some were one-off research studies, as in the UK). Italy and Spain are the only Southern European countries which have reported any ASD screening experience (ongoing health surveillance programmes in both cases). Belgium is the only country where the screening study was set in child day-care centres rather than in primary care. Five countries have used or are using the M-CHAT as their screening instrument of choice (sometimes together with another ASD screening tool). A contemporary map of Europe with the information compiled through the ESSEA COST network in 2012 is depicted in Fig. 2.

Through the ESSEA-COST network, we also gathered first-hand information about ASD screening in Norway. The Autism Birth Cohort (ABC), a sub study of the Norwegian Mother and Child Cohort Study (MoBa) has included several ASD checklists on the 18-month questionnaire, i.e. M-CHAT, ESAT and the Non-Verbal Communication Checklist (NVCC) (Schjolberg, submitted). At age 36 months, the 40-item Social Communication Questionnaire (SCQ) has been used to screen for ASD in the complete MoBa cohort (*N* ~ 100,000). Screen-positive children underwent a full-day diagnostic evaluation using ADI-R and ADOS. The entire MoBa cohort is followed up at 8 years with the complete SCQ enabling researchers to examine ASD symptom patterns from early age to 8 years. Linkage to the Norwegian National Patient Registry (NPR) makes it possible to identify false negatives from the early screening. This study represents the largest sample of children screened for ASD in Europe (approximately 100,000), though it is not an ASD screening programme per se and indices on the screening tools are not yet published. The study is described in Stoltenberg et al. [65], and the relationship between screen positivity at 36 months and subsequent ASD diagnosis at assessment are being prepared for publication (Bresnahan et al., in prep.). Beuker et al. [66] have examined whether ASD symptoms in 18-month-old children fit the 3-factor structure, as described in DSM-IV. Characteristics of M-CHAT at 18 months compared to later diagnostic status based on clinical assessment or NPR (ASD vs non-ASD) are in preparation for publication (Stenberg et al., in review).

A second reading of the full text of the selected papers was completed by the main authors of this paper (PGP and AH). Study methodologies were thoroughly reviewed to identify differences among screening procedures, as well as the main factors that might influence screening programme results. As a result, a list was drawn up containing ten critical factors to be considered when assessing screening studies. To contextualise these factors, additional information from both European and non-European studies was included, where appropriate.

## Factors to be considered when evaluating screening studies

The ten factors to be borne in mind when assessing screening studies are: (1) broad-based analysis of validity indices; (2) prevalence rates and PPV interpretation; (3) age of screening; (4) level of functioning and autism severity; (5) selection and formulation of items; (6) cut-off criteria; (7) protocol adherence; (8) informants; (9) parental non-compliance rate; and (10) setting characteristics: organisation of services, as shown in Table 3. Each of these methodological issues will now be addressed in turn.

**Table 3:** Factors to be considered when evaluating screening studies

Factor	Key description
I. Broad-based analysis of qualitative indices	Need for comprehensive approach and consideration of intervention benefits of FP cases besides possible side effects
II. Prevalence rates and PPV interpretation	“Population-based” sample vs. “High-risk” sample
III. Age of screening	Younger age ≥ higher FP rate; difficulties in differentiating “ASD” from “other DDs”
IV. Level of functioning and autism severity	Higher IQ and/or milder variants of ASD ≥ higher FP rate
V. Selection and formulation of items	Specificity: play + sensory + motor skills (young age); social interaction and communication (older age); importance of formulation: ever vs. rarely
VI. Cut-off criteria	Importance of exploring different cut-off scores for different purposes and populations
VII. Protocol adherence	Lack of consistency of screening procedures across studies. Need for balance between protocol adherence and deviations, depending on study purpose/resources
VIII. Informants and training	Parents, paediatricians, primary care physician, child care workers and child nurses. Good training programmes together with the tool
IX. Parental non-compliance rate	Socio-economic, ethno-cultural and age-related factors. Importance of re-test
X. Setting characteristics: organisation of services	Challenges of each screening context. Importance of availability and coordination between related services (i.e. screening, diagnostic and intervention services)

Justification for/discussion of these ten factors also considers literature from non-European studies

### Broad-based analysis of validity indices

Studies report several parameters that assess the efficacy of screening instruments. Sensitivity and specificity are often considered the most important criteria of validity. A major challenge, however, is the interpretation of these values. Although interpretation is facilitated by the establishment of quantitative criteria, with values of 0.70 or higher being acceptable for developmental disorders [67], a more comprehensive approach to interpreting these parameters is called for. A trade-off between sensitivity and specificity is common. A screening procedure with a high sensitivity will often have a high false-positive rate, thereby lowering its specificity. Screening methods with a high specificity will usually sacrifice sensitivity by increasing the false-negative rate. This is also demonstrated by some of the screening procedures in Table 2. For instance, the CHAT-1 + CHAT-2 (second administration of CHAT after a high-risk result in CHAT-1) has an excellent specificity of 1.00 combined with a very poor sensitivity of 0.21 [62]. It has been suggested that sensitivity is the measure of greatest concern [68, 69]. The drawback of many false negatives (low sensitivity) is that many children who will go on to develop ASD are missed. This precludes early diagnosis and early initiation of treatment and family support for such children and their families. On the other hand, a low specificity also has negative implications. False positive cases are evaluated through costly assessment procedures, not to mention the possible stigmatisation of the child and the additional family stress caused by falsely alarming parents [70]. These consequences resulting from an erroneous positive identification could be considered as negative side effects of a screening programme with insufficient specificity. However, when interpreting the false-positive rate, it is crucial to consider the proportion of false-positive cases that have another developmental delay or disorder. Dietz et al. [43] reported that 25 % of all ESAT false-positive cases had a language disorder, and 18 % of the false-positive cases were diagnosed with intellectual disability. These findings raise questions as to whether screening procedures should target ASD specifically or developmental disorders and delays in general [70]. Instead of immediately rejecting a screening procedure with a high false-positive rate, a more in-depth look may indicate that the screening procedure is helpful in detecting children who benefit from further diagnostic assessment and treatment at an early age. The amount of false-positive cases having another developmental disorder justifies the need to examine developmental trajectories, to gain insight into which early signs are specific for ASD [71]. Performing screening through a two-stage process before any diagnosis referral (which is characteristic of most procedures in Table 2) may help to narrow down false-positive rate and thereby reduce the above-mentioned possible side effects of screening.

### Prevalence rates and PPV interpretation

A high degree of variation in ASD prevalence has been reported. Age, diagnostic criteria and region have been found to be associated with ASD prevalence rates [72]. Although the PPV is often considered to be the most useful information for the clinician [73], its value depends on the prevalence rate in the population screened. This might explain why the PPV was lower in the Spanish M-CHAT study than in other M-CHAT studies [44]. The frequency of ASD cases observed in the Spanish study (0.92 % in Stage 1; and 0.29 % in Stage 2 based only on a general population sample) was much lower than that reported by other M-CHAT studies (e.g. 2,7 % in Kamio et al. [74]; 2.66 % in Robins et al. [19], 3.03 % in Kleinman et al. [75] and 2 % in Pandey et al. [76], in which most ASD cases came from their referred early intervention sample rather than from their general paediatric practices. These considerations highlight the importance of knowing the prevalence of ASD in the population targeted for screening, instead of relying on the PPV reported by another study with another prevalence rate in, say, a different age range [77]. One method for calculating the validity of a screening instrument which takes into account ASD prevalence is Bayes Theorem. According to this theorem, the chance of a disease being truly present depends on both the prevalence of the disease and the properties of the test, essentially the likelihood ratio [77, 78]. Rather than using the prevalence in a specific sample, e.g. by examining the clinicians’ records within that specific context, as recommended by Camp [73], some authors have instead used prevalence rates drawn from a different sample to estimate validity properties [73]. For instance, Groen et al. [78] used the prevalence rates reported by Baird et al. [62] to evaluate the validity of the ESAT [78]. When clinicians use these numbers to support their choice of the ESAT, it should be borne in mind that there might be a difference in prevalence rates between populations. This underscores the importance of clarity as regards the prevalence rate used in validity studies and the usefulness of pre-test odds and likelihood ratios. Since prevalence of autism in the general, unselected population is very low [79], Groen et al. [78] suggest that one possibility of increasing the post-test odds is to increase the pre-test odds by applying screening instruments solely to selected children who are either found to have a deviant developmental path in routine developmental surveillance, or found to have high-risk status by other means.

### Age of screening

Several studies show that parents have concerns about children who later develop ASD within the first 2 years of life: De Giacomo and Fombonne [80] report an average age of 19 months, while Chawarska et al. [81] report an age of 14 months. In the presence of intellectual disability, an older sibling, concerns for medical problems, or a delay in developmental milestones, the age of parental concern were lower [80]. Yet, detecting ASD at a very early age is not exempt from considerable difficulties, since it may be difficult to differentiate ASD from other developmental disorders [82], or even to differentiate ASD from typical development [83]. For example, repetitive behaviours are also present in young children with typical development [84]. Moreover, many behaviours that capture joint attention skills, such as gaze monitoring and protodeclarative pointing, develop gradually from age 9 to 18 months in typically developing children [85], and are only a clear clinical sign when they have not appeared after the age of 18 months. Difficulties in differentiating ASD from other developmental disorders at a very early age are consistent both with findings from the ESAT screening at 14 months which resulted in a high number of false positives, though none of these children had typical development [43], and with the CESDD study [24]. Dereu et al. [86] report many false positives, specifically in the younger age group. Moreover, the false-negative rate might also be higher at a young age due either to late onset of ASD or to the fact that about 30 % of children show regression after a period of typical development [81]. It is also plausible that milder variants of ASD, and children with a higher level of cognitive development could be missed at a young age [43]. Thus, when interpreting validity indices, it is important to consider the age of the sample during screening and diagnostics.

### Level of functioning and autism severity

Since ASDs are associated with a broad range of intellectual and language skills that change over time, level of functioning and autism severity are important factors to consider when evaluating screening methods. Children who were not identified by the CHAT but were later diagnosed with ASD were found to be higher functioning in a variety of areas and were rated as less severe on autism assessment measures [87]. A study by Kleinman et al. [75] showed that this was similar for M-CHAT, with false-negative cases being higher functioning than positive M-CHAT ASD cases. The SCQ showed better discriminative validity in toddlers with intellectual disability than in those without intellectual disability, and also showed that IQ significantly predicted SCQ scores [88]. This may reflect the fact that higher functioning toddlers with ASD are more difficult to distinguish from their high-risk, non-spectrum peers than are low functioning toddlers. Since screening instruments are intended for broad use, an effect of IQ is a problem. In a different study, Oosterling et al. [88] reported that, after a screening procedure with the ESAT, about 75–85 % of the children referred before 36 months with narrowly defined autism had intellectual disability. Difficulties in screening for ASD in young children, and difficulties with diagnostic discrimination in high-risk children in particular, are issues that are not necessarily specific to the screening tool, especially with regard to specificity, but rather to the IQ or risk status of the children [88]. Hence, clarity regarding the characteristics of the sample used is very important when interpreting the psychometric properties of the instruments under investigation.

### Selection and formulation of items

ASD screening procedures vary in the items included to identify children at risk. Social-communicative impairments are considered to be central to ASD [52] and are therefore always part of screening procedures. The item ‘lack of following joint attention’ was indeed one of the items that was most effective in distinguishing ASD from non-ASD cases when using the CESDD [88], and the CHAT mainly consists of items on initiating and following joint attention [16]. Social-communicative items in the ESAT, including ‘shows interest in people’, ‘smiles directly’ and ‘reacts when spoken to’ also discriminated best between children with and without ASD [43]. Even so, many studies have shown that screening procedures which focus exclusively on social-communicative impairments might overlook other early signs of ASD. In a familial, high-risk sibling sample, Zwaigenbaum et al. [4] showed that early behavioural markers for ASD include atypical markers in visual tracking, disengagement of visual attention and sensory-oriented behaviours. Gillberg [89] reported that ‘does not play like other children’ was among the three most discriminating items and further suggested that abnormal perceptual responses are important for identification of ASD. Other studies have supported the existence of abnormalities in play and sensory-motor behaviours at an early age [3, 90]. The results of these studies have broadened the focus of screening instruments for ASD, and this has been effective. Among the items with the highest odds ratio in the CESDD study were ‘lack of symbolic play’ and ‘unusual sensory behaviour’ [24]. In addition to the CESDD, many other screening instruments (ESAT and M-CHAT) have included items focused on play and sensory-motor behaviours. Baird et al. [62] suggest that specifically the combination of failing joint attention and pretend play at 18 months indicates risk of developing ASD. The fact that sensory and motor items have not been included in all screening tools might be due to the fact that parents do not mention these items spontaneously. However, when parents have been questioned about these items specifically, they report having noticed such abnormalities from an early age [22]. At a young age it might be useful to take play-related behaviours into account, while at an older age, impairments in social interaction and communication might become more specific behavioural markers for ASD. In the ESAT study some items, such as ‘gaze following’, had a relatively high proportion of negative answers for children younger than 12 months because this trait is still developing in the first year of life [43]. For instance, the First Year Inventory (FYI) [23] developed to assess behaviours in 12-month-old infants and the ESAT [43] developed for 14-month-old infants include more play-related and sensory-motor behaviours than does the SCQ [91], which was originally developed for individuals aged 4 years and over. On the other hand, the SCQ includes items such as ‘pronoun reversal’, ‘verbal rituals’ and ‘no friends’, which are more appropriate for somewhat older children. Differences in the formulation of items might also affect the responses. The CESDD, for example, includes the item ‘lack of showing objects to others to indicate interest’, which was recognised in 64.52 % of children with ASD. In contrast, the item ‘absence of showing’ in the ESAT and M-CHAT was recognised in only 26.67 and 28.57 % of children with ASD, respectively, while ‘no showing’ in the SCQ was recognised in only 13.04 % [86]. Baird et al. [62] also point to the fact that in the CHAT parents were asked to report whether their child had ‘ever’ produced certain behaviour, while if they had been asked if their children had only ‘rarely’ produced such behaviours, the instrument’s sensitivity might have been higher, though at the cost of its PPV and specificity.

### Cut-off criteria

Instead of continuing to develop new screening methods for ASD, a more elaborate evaluation of current screening methods might be helpful. One way of achieving this is to explore different criteria within the same screening procedure, using different cut-off scores for different purposes and populations. Comparing the validity indices of the CESDD in combination with an SCQ cut-off of 11 to those of the CESDD in combination with an SCQ cut-off of 15 demonstrated that lowering the SCQ cut-off to 11 improved sensitivity from 0.42 to 0.70 while maintaining good specificity (Dereu et al., unpublished data). Oosterling et al. [63] also explored different criteria of the SCQ (cut-off 11 vs. 15) and the CHAT (high or high + medium risk considered positive). This study showed that, whereas sensitivity was higher for the SCQ cut-off of 11 as found in Wiggins et al. [92], specificity was higher for the SCQ cut-off of 15. In the case of CHAT validity, the high-risk + medium-risk criterion improved sensitivity considerably (from 0.18 to 0.48) while keeping specificity high, i.e. 0.99 for the high-risk criterion and 0.87 for the high-risk + medium-risk criterion. In addition, Scambler et al. [87] described how a slight change in CHAT criteria to allow parents to endorse either of two critical items, improved CHAT sensitivity by 20 % while maintaining specificity of 100 % in a group of children with developmental disabilities. In the Spanish M-CHAT study, false-positive cases were found to be reduced if the M-CHAT was only deemed to be positive after five [44] as opposed to three failed items [19].

### Protocol adherence

Another factor that may cause variation in screening results is the fact that the same screening procedure is often implemented in different ways. Administration is not consistent across different studies. Researchers and clinicians adapt the original protocol of the screening procedure to their own needs and circumstances. The M-CHAT, for instance, comprises a 23-item yes/no parent report and a follow-up telephone interview. This interview was added to the initial M-CHAT protocol to reduce the number of false positives [19]. Kleinman et al. [75] found that by adding a telephone interview to the screening procedure, the PPV was improved from 0.36 to 0.74. This was especially important in the low-risk general population. Both Nygren et al. and Canal-Bedia et al. [27, 44] indicate that the interview is necessary because items are sometimes misunderstood. Although adding the phone interview proved effective, it should be noted that some researchers have adapted this procedure. Dereu et al. [86] did not include the telephone interview, so that positive screens on the M-CHAT were based exclusively on parent report. This may have affected the PPV, which was 0.29, for the procedure, which consisted of the CESDD with the M-CHAT but without the telephone interview. In some cases, however, it may be more effective to forget the interview. In a case where children fail seven or more items in M-CHAT initial screening, a follow-up interview may not be necessary [93]. Such children can be immediately referred for further evaluation. An alternative way of conducting the follow-up interview is to be seen in Spain, where the M-CHAT interview is computer-based and performed directly by the paediatrician after a positive result, by asking the parents about the failures, an approach that obviously facilitates administration of the follow-up process [64] or implementing the M-CHAT entirely in electronic format [94]. Another example of alternative administration can be found in the study by Oosterling et al. [63]: instead of using the CHAT as a separate instrument, items from the SCQ and CSBS-DP were combined to represent CHAT items, which probably influenced the results. When implementing a study protocol, adherence and deviation should be balanced, bearing in mind the specific purpose and resources of the study. It needs to be specified here that a revised version of the M-CHAT (M-CHAT-R/F; [95]) with an algorithm based on three risk levels has been recently published and recommended for primary care settings.

### Informants and training

The information extracted from the studies reviewed shows that many different informants are used in ASD screening. Filipek et al. [96] noted that parents are often correct in their concerns about their child’s development. Although parents may not be as accurate when it comes to specific ASD deficits, they are almost always accurate in detecting a developmental problem [67]. Since parental checklists, such as the M-CHAT, are easy to administer, they are often used for screening purposes. Yet, parents may not know exactly what skills to expect at a certain age and are not able to compare their child with peers [86]. Furthermore, parents may also over- or under-report problems in their child. In the ESAT study [43], ASD experts evaluated children’s behaviour more negatively than did their parents, to the extent that 3 out of 18 children diagnosed with ASD would have scored below threshold on the 14-item ESAT if only parent rating had been used. Accordingly, parental information should be combined with observations by a professional, such as a physician. Physicians, and paediatricians in particular, possess knowledge about typical child development [88, 97] and are able to compare the behaviour of the child to that of his/her peers. It should be noted, however, that physicians have to base their clinical judgment on a brief observation of the child and a short conversation with the parents. Moreover, the behaviour of the child when examined by the physician or another clinician may not represent the child’s typical behaviour in a natural context. To prevent the problems posed by only parents’ or physicians’ reports, child care workers might also be very useful as informants; since they can compare behaviour and the development of the child directly to that of other children and are educated in typical development. In addition, children may behave more typically in a child care setting than at a medical practice, since children often visit child care on a regular basis [24].

Other authors have also suggested the possible contribution of child care workers to ASD screening in young children [98]. In the UK, the NICE guidelines recommend training professionals in early signs of ASD at pre-school and school ages [99]. It is important to understand that training physicians and professionals in recognising early signs of ASD might make a crucial difference in the results of screening. The DIANE Project in The Netherlands [88] is a good example of health care professional training, in which small groups of primary care workers attended a compulsory course of interactive training sessions. The main part of the training sessions included a review of early signs of autism and all ESAT items, illustrated by video clips showing children with abnormal or absent behaviour and others showing typically developing children, to clarify what could be expected of a young child at a certain age. In general, the results of this controlled study support the fact that the availability of an early identification tool, coupled with training for primary care workers in the early signs of ASD and their ongoing involvement in a screening programme can lead to earlier detection, referral and diagnosis of ASD. Lack of training could lead to disagreement over ‘cookbook’ guidelines, unfamiliarity with screening instruments and procedures, as well as inconsistent knowledge of ASD and fear of positive results among primary care providers [88].

### Parental non-compliance rate

Parental non-compliance is an essential problem in many screening studies. It is, therefore, imperative to examine the differences between parents who are compliant and non-compliant with the screening instrument and to provide explanations for non-compliance. Firstly, parents are known to be more inclined to participate in cases where the atypical development of their child is more apparent. Screening scores have been shown to be higher in the children of compliant parents than in those of parents who declined further assessment [43]. Secondly, children of compliant parents were somewhat older at the time when their parents completed the questionnaire [86]. This may be related to the above factor. Parents may not comply because they do not have any concerns about the development of their child at very young ages, or alternatively, because the symptoms may not yet be apparent at this stage [43]. A possible solution could be to ask parents again the following year when their child is slightly older, something that may serve to increase the response rate. Dereu et al. [24] suggest that a more personal approach might improve parental compliance. This might explain why the response rate was lower for returning parent questionnaires than for further developmental assessment [24]. Another factor to facilitate compliance might be to limit the number of assessments requiring parents to come in person to the university or health centre with their child. In the study by Dietz et al. [43], the effort of undergoing a minimum of two, but preferably, five examinations at the department was an important obstacle to participation. Dereu et al. [24] also report that parents did not wish to subject their child to the burden of assessments, and for some parents it was just not feasible to come to the university. Socio-economic and ethno-cultural factors may also have an effect on compliance, i.e. Reznick et al. [23] report that Afro-American families and less-educated parents more often refuse to participate. One reason for this might be the fear of the stigma attached by some cultural groups for receiving a diagnosis [100].

### Setting characteristics: organisation of services

*A* screening procedure cannot be implemented without taking the setting characteristics into account. The presence of a preventive health system, such as the well-baby clinics in The Netherlands and the well-baby check-up programme in Spain, offers the opportunity to screen at a population level as opposed to screening high-risk children alone [43, 44]. One advantage of the presence of such a system is also the high attendance rate, often related to compulsory vaccinations. Even where such a system is available, it is still relevant to examine whether the system is available to all residents and whether it covers families from all socio-economic and ethno-cultural groups. Canal-Bedia et al. [44] also note the need for coordination between the health system and early intervention units in Spain. Needless to say, when implementing a screening procedure, post-screening intervention in the form of diagnostic assessment and intervention programmes should also be made available. Coordination with such services is also crucial for identifying possible false-negative cases [64]. Another factor to be considered is that there might be many differences in physician training and education in the respective countries. This is something that should be assessed when implementing a screening procedure which relies on physicians as informants. In addition, when choosing the CESDD as a screening procedure, it is important to bear in mind that this instrument might not be as effective in countries where only few children attend child care facilities, either because of the expense involved or because only a minority of women work. Child care in such countries might also be provided by the extended family instead of professional child care workers. In these cases it might be better to choose another procedure, since the CESDD’s advantages (i.e. the ability of child care workers to compare the child’s development to that of peers) are not applicable.

## Other methodological concerns about ASD screening studies

A major issue in studies that evaluate the validity of ASD screening procedures is that not all children were followed up. In particular, information on screen-negative cases is missing in many screening studies in Table 2. Some studies have attempted to ‘solve’ this problem by calculating the sensitivity and specificity based on general prevalence rates, e.g. Groen et al. [78] calculated validity indices for several screening instruments, using ASD prevalence numbers reported by Baird et al. [62]. As mentioned earlier, however, the prevalence rates of the populations studied may differ, particularly as prevalence estimates are age dependent, since some children might not clearly manifest the full range of ASD symptoms until social demands outstrip capacity, as recognised by the new DSM-5 diagnostic criteria [101]. Oosterling et al.’s study [63] reported sensitivity and specificity based on the percentage of children who had already been the focus of some concern about ASD, a very specific group: true validity indices cannot be ascertained in this case. Future studies should devote more effort to the follow-up of screen-negative cases to calculate the true validity indices in that specific sample, though it should be noted that following up such cases could be expensive since a majority may prove to be genuinely screen negative [44]. On the other hand, it is plausible that some screen-negative cases will receive a diagnosis. Higher functioning children, children with less severe autism, and children who exhibit regression have a high probability of being missed in screening procedures [96]. Extending the inclusion criteria by, say, also including children who fail language items may improve estimates of validity indices by detecting false-negative cases (Dereu et al. [24]). It is likewise important to continue monitoring screen-positive cases, to establish the validity of the screening procedure in terms of a clinical diagnosis over a longer period of time. For screening studies it is critical that the follow-up of children be envisaged in advance. This idea has also been supported in a recent study examining over twenty different ASD screening programmes in the USA. One of main conclusions is the importance of methodological rigour and the quality of measures in the screening studies [51]. In the CHAT study, only half the children in the medium-risk group were not further evaluated due to lack of resources [62].

In addition, future studies should be designed in such a way that makes it possible to examine the influence of sample-specific factors on screening results. Thus, a sample should include different age, socio-economic and ethno-cultural groups. Similarly, the study population should preferably include children across the whole range of intellectual functioning. Although this was done in the ESAT studies (Dietz et al. [43]), the original CHAT study excluded children with a clear developmental delay (Baird et al. [62]). Some studies did examine the influence of sample-specific factors on sensitivity and specificity, by examining the screening results for specific age, IQ and diagnostic group [62, 68]. In general, a sample size should also be large enough to ensure that the validity indices of a screening method can be reliably calculated.

## Conclusions and implications for future research

The aim of this review was to provide an overview of the screening procedures that have been evaluated in research studies across Europe, and the issues and methodological concerns associated with these. Currently, only the screening procedure with M-CHAT in Spain is still being used in routine practice. The other screening instruments that have been evaluated in research studies, such as the ESAT and the CESDD, are available for use by professionals but are not part of routine practice.

We trust that this analysis will, not only inform the drafting of recommendations for early identification of ASD, but will also prove especially important to European countries with no experience in ASD screening when it comes to making the correct choices about how to implement a screening programme in a specific setting.

Although there is consensus on the importance of early detection from both a research and clinical point of view, choosing a screening procedure that fits a certain context may be still difficult. This choice has to be based on arguments beyond validity indices. As this review has shown, findings regarding screening should be interpreted with caution. It is critical that clinicians understand how to interpret data from published studies [102]. It should be noted that screening outcomes are influenced by several factors. Therefore, a more expansive and balanced way of evaluating screening methods, which takes into account all the factors that may influence the results of the screening, is recommended. In addition, methodological issues should also be considered. The fact that in many studies screened-negative cases are not followed up, may have distorted screening outcomes. It is important to identify missed cases. This may be done by longitudinal population studies which screen children from an early age until an age at which ASD is likely to be detected or is, at least, likely to be detected with a second measurement at a later age [75]. However, due to parental non-compliance and limited resources, this is often difficult to achieve [62, 75]. Screening information should be carefully communicated to parents [102]. The need of motivational strategies to ensure that families will participate longitudinally and will follow-up treatment recommendations has also been highlighted in recently published manuscripts. They support the usage of rigorous methodology and evaluation of further variables when screening, such as rates of referral and uptake of services which have been rarely documented in screening studies [51, 103].

In USA, M-CHAT-R/F has demonstrated to be an effective tool for screening low-risk toddlers, reducing the age of diagnosis by 2 years [95]. New possibilities stimulated by these findings could be assessed towards widespread ASD screening in Europe. Recent recommendations from American Academy of Child and Adolescent Psychiatry (AACAP) maintain the support to ASD screening to young children and in some instances also relevant to older children [104]. There are also now new doors opened with concrete suggestions about how to conduct cluster randomised trials of ASD early screening [105].

Our review has attempted to analyse the current situation of early detection of ASD in Europe. Although the issues surrounding screening are relevant for any screening procedure to be implemented in Europe and beyond, greater in-depth knowledge of inter-country differences is still required. The diversity in government policy, health care, educational, and social-care settings and cultures across Europe means that screening procedures cannot be fully standardised. Joining efforts towards screening populations in lower income countries that usually access later to the intervention services should be prioritised. For instance, a preventive care system with a high attendance, such as the well-baby clinic, may not be available in every European country, making it more difficult to implement routine developmental surveillance. Thus, implementation of routine screening for ASD and/or other developmental disorders may require a reorganisation of the health care system in many countries. Screening is only effective for clinical purposes when diagnostic centres and interventions are also available.

A detailed characterisation of the samples of participants in the different screening studies, taking into account important variables such as ethnicity and socio-economic status, is needed if further conclusions are to be drawn. Additionally, a pooled data analysis of the items shared by the different screening instruments used in the European context aims to yield interesting results (Maganto, in prep).

At the moment, as part of this ESSEA-COST Action, one of the four working groups (WG3: testing how well screening instruments work in prospectively identifying cases [47]) is carrying out ongoing survey whose main goal is to compare the current status of early developmental surveillance across the 28 Member States of the European Union. Thus far, over 17 countries have responded, including at least two different informants per country. The information collected will, not only show how ASD detection and diagnosis is approached in each country, but will also provide objective data for calculating screening programme performance indicators in those countries where a system for early detection of autism exists or has existed as compared to those where no such system is or has ever been in place.

To date, a wealth of ASD screening procedures is available in Europe. While knowledge is shared through international publications and conferences, collaborations, such as the ESSEA COST Action Network, contribute to sharing knowledge among researchers and clinicians in a more direct way. Future challenges for this network lie in raising awareness about early signs of ASD among parents, child care professionals and physicians across Europe, evaluating and adapting the use of current screening procedures for different countries, providing an accessible platform for sharing knowledge and resources among European researchers and clinicians, and, most importantly, improving developmental outcomes for children with ASD and their families. Notwithstanding encouraging experiences, there is still much to be done.
